# Optimization of Direct Aromatic ^18^F-Labeling of Tetrazines

**DOI:** 10.3390/molecules27134022

**Published:** 2022-06-22

**Authors:** Ida Vang Andersen, Rocío García-Vázquez, Umberto Maria Battisti, Matthias M. Herth

**Affiliations:** 1 Department of Drug Design and Pharmacology, Faculty of Health and Medical Sciences, University of Copenhagen, Jagtvej 160, 2100 Copenhagen, Denmark; ida.andersen@sund.ku.dk (I.V.A.); rociogv@sund.ku.dk (R.G.-V.); umberto.battisti@sund.ku.dk (U.M.B.); 2 Department of Clinical Physiology, Nuclear Medicine & PET, Rigshospitalet, Blegdamsvej 9, 2100 Copenhagen, Denmark

**Keywords:** tetrazine ligation, pretargeted imaging, fluorine-18, copper-mediated fluorination

## Abstract

Radiolabeling of tetrazines has gained increasing attention due to their important role in pretargeted imaging or therapy. The most commonly used radionuclide in PET imaging is fluorine-18. For this reason, we have recently developed a method which enables the direct aromatic ^18^F-fluorination of tetrazines using stannane precursors through copper-mediated fluorinations. Herein, we further optimized this labeling procedure. 3-(3-fluorophenyl)-1,2,4,5-tetrazine was chosen for this purpose because of its high reactivity and respective limited stability during the labeling process. By optimizing parameters such as elution conditions, precursor amount, catalyst, time or temperature, the radiochemical yield (RCY) could be increased by approximately 30%. These conditions were then applied to optimize the RCY of a recently successfully developed and promising pretargeting imaging agent. This agent could be isolated in a decay corrected RCY of 14 ± 3% and Am of 201 ± 30 GBq/µmol in a synthesis time of 70 min. Consequently, the RCY increased by 27%.

## 1. Introduction

Tetrazines (Tzs) have gained great interest as radiotracers in nuclear medicine, as a consequence of their versatility for pretargeting, but also as a synthon to label nanomedicines orthogonally [1,2,3]. The Inverse-Electron-Demand Diels–Alder (IEDDA) reaction between tetrazines (Tzs) and trans-cyclooctene (TCO), is currently the reaction that offers the most powerful features for click chemistry [4,5]. This is because of its unique selectivity and additionally, because of its fast reaction kinetics, which are up to five orders of magnitude higher than the strain-promoted alkyne-azide cycloaddition reaction (SPAAC) and up to seven orders of magnitude higher than the Staudinger–Bertozzi ligation [6,7,8]. Therefore, and in addition to conventional labeling approaches, the use of Tzs as radioligands for pretargeting has emerged as a new powerful technology [9]. In pretargeting, a non-radiolabeled but TCO-tagged targeting vector is administered and allowed to accumulate at the target for several days before injection of a radiolabeled Tz. This Tz will then rapidly and specifically click with the TCOs tagged to the targeting vector. In this way, the selectivity and affinity of monoclonal antibodies (mAbs) and the good pharmacokinetics of small molecules can be combined [4,10]. This approach can be used to obtain images of nanomedicine with better contrast at earlier time points. Another interesting use of radiolabeled Tzs—as mentioned before—is the possibility of using these agents to label sensitive compounds under mild physiological conditions [11,12,13]. For these reasons, several radiolabeled Tzs have been reported labeled with ^11^C, ^18^F, ^68^Ga, ^64^Cu or ^177^Lu [11]. Fluorine-18 is the most widely used radionuclide for clinical applications, because of its nearly ideal characteristics: a good branching ratio (96.7% β^+^), a short positron range (2.4 mm max. range in water), and a short half-life of approximately 110 min, resulting in high-resolution PET images, good distribution range and scalability and additionally, an acceptable radiation dose. For these reasons, the labeling of Tzs with fluorine-18 has recently received increasing attention [14,15,16,17].

Direct labeling of sensitive compounds can sometimes be challenging because of the harsh reaction conditions required for ^18^F-fluorinations [18,19]. For example, highly reactive mono- or bis-(hetero)aryl-substituted Tzs tend to decompose under the nucleophilic conditions used for standard ^18^F-fluorination approaches (S_N_2 or S_N_Ar) [15,17,20]. As a result, many efforts have been made to improve radiolabeling conditions so that these methods can be applied to compounds that could not previously be ^18^F-fluorinated [18,21,22]. Mossine et al. recently reported a ^18^F-labeling method using relatively mild labeling conditions for copper-mediated fluorination of tin precursors [18]. This method allowed for the use of non-nucleophilic bases for the elution of fluoride-18 from anion exchange cartridges (fluoride-18 is standardly trapped on these cartridges during its work-up), and reduced the basicity of the reaction [18]. Our group has advanced this methodology for the ^18^F-labeling of highly reactive Tzs [17,21,23,24,25]. In particular, we reported the first direct aromatic fluorination of Tzs from stannane precursors [23]. We were also able to automate the reaction—a main requirement for the implementation of radiolabeling procedures for cGMP production of clinically relevant PET probes [19]. Additionally, aromatic ^18^F-tracers are typically more stable towards defluorination than their aliphatic counterparts [26,27,28]. 

**Figure 1:**
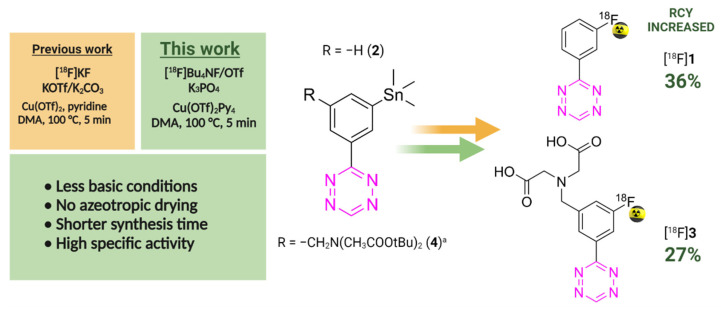
Overview of this work. Previous approach (orange); the new approach reduces the amount of base needed, removes the azeotropic drying step, results in a shorter synthesis time and higher RCY (green). ^a^ The reaction proceeds in a two step, one pot sequence.

In this work, we optimized this Cu-mediated ^18^F-labeling procedure by exploring several parameters such as the influence of preconditioning and elution conditions, the variation of the precursor and catalyst amount, and the influence of reaction time and temperature on the radiochemical yield (RCY) (Figure 1 and Table 1). All these parameters have been shown to influence the yield of ^18^F-fluorination procedures [21,23]. 

## 2. Results

Table 1 summarizes the parameters that have been optimized within this study to improve the RCY of Tzs. Only one parameter at the time has been explored. As a starting point, we decided to use the conditions that have been reported as resulting in the highest RCY for these kinds of reactions. These conditions are highlighted in italics in Table 1. We decided to use 3-(3-fluorophenyl)-1,2,4,5-tetrazine (**1**) as a model compound for our optimization attempts. Model compound **1** was selected due to its accessibility, relatively high reaction kinetics (67,000 M^−^^1^ s^−^^1^ in DPBS, 37 °C) and limited stability against temperature and nucleophilic attack. Because of these properties, we believed that **1** was an optimal scaffold to study this type of reaction, [23] as the parameters result in a compound that represent Tzs that are difficult to label. Labeling conditions that work for **1** should be transferable to all other Tz structures. 

### 2.1. Influence of the Preconditioning and Elution Conditions

First, we investigated the influence of the preconditioning and elution conditions of the used anion exchange cartridge to trap and release [^18^F]fluoride delivered from the cyclotron. These parameters are crucial as low trapping efficiency and low elution of [^18^F]fluoride translate to low yields even if the reaction itself possess a high radiochemical conversion (RCC) [21]. The preconditioning and elution conditions tested are listed in the supporting information (SI, Table S1) [24]. 

**Figure 2:**
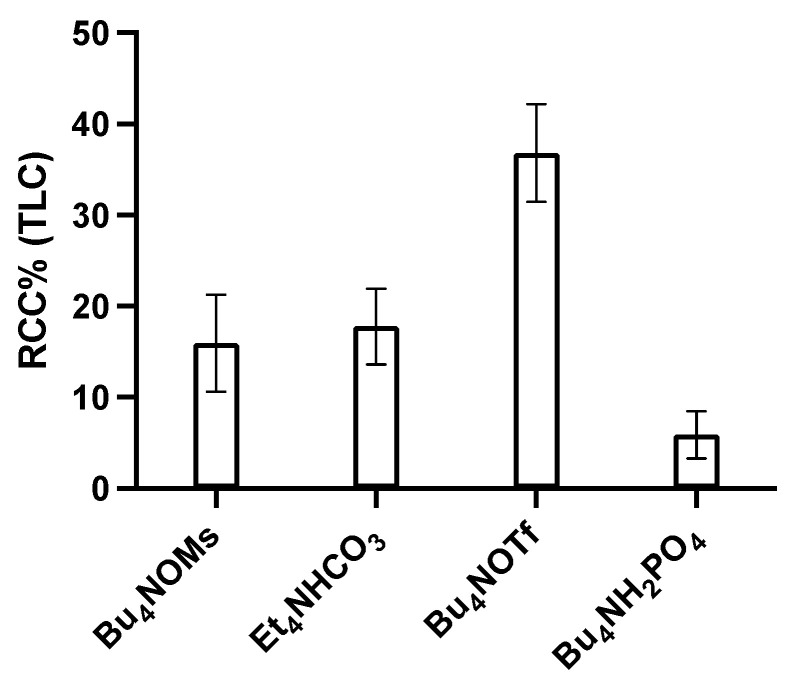
Effect of different elution solutions on the RCC. Reaction conditions: QMA preconditioned K_3_PO_4_ 0.5 M, [^18^F]fluoride (~50 MBq) loaded, flushed with 0.5 mL MeOH and eluted with one of the eluents shown in the x-axis (20 mM) in MeOH 1 mL. Dried under nitrogen gas for 5 min at 100 °C before adding precursor 3-(3-trimethyltin)-1,2,4,5-tetrazine (**2**) (3.2 mg, 10 µmol) and Cu(OTf_2_)Py_4_ (10 mg, 15 µmol) in 0.5 mL DMA, 5 min at 100 °C. The crude reaction was analyzed by radio-TLC and radio-HPLC (SI, Table S1, Figures S1 and S2). *n* = 3, error bars = standard deviation.

Different eluent systems were tested, at a concentration of 20 mM (in MeOH, 1 mL), to explore their influence on the elution efficiency (EE) and RCC of the reaction. We tested Bu_4_NOMs, Et_4_NHCO_3_, Bu_4_NOTf, Bu_4_NH_2_PO_4_, and TEAHF. Besides the latter elution system, the elution efficiency (EE) exceeded 90% for all other conditions when the QMA was preconditioned with 0.5 M K_2_PO_4_ (aq.). In contrast, the observed RCC differed depending upon which elution system was used. Radiolabeling using Bu_4_NH_2_PO_4_ as eluent resulted in RCCs of 6%. This could be due to the acidity of the eluting anion. As expected, elution with the non-basic anions, OTf^−^ and OMs^−^, as well as using Et_4_NHCO_3_ in combination with multicharged phosphate (PO_4_^3−^) for precondition the QMA resulted in higher RCCs (Figure 2). The highest RCC was achieved using Bu_4_NOTf and can be explained by the lower pKa value of OTf^−^ compared to the other anions used. This translates to a lower ability of OTf^−^ to displace preconditioning anions during the elution. Consequently, less basic conditioning anions are eluted and the basicity for subsequent reactions in the reaction vial is lowered.

Subsequently, preconditioning with other anions such as K_2_HPO_4_ (aq.), KOTf (aq.) and K_2_CO_3_ (aq.) was tested, while the eluent was fixed to Bu_4_NOTf (20 mM in MeOH). KOTf precondition in combination with Bu_4_NOTf, gave low elution efficiency (EE) of ^18^F-fluoride (SI, Table S1). Preconditioning with K_2_HPO_4_ and KOTf resulted in lower RCC (0–1.8%) of the reaction. On the other hand, previously reported conditions where KOTf is used for precondition, in combination with both OTf^−^ and CO_3_^2−^ anions for the elution of the QMA, afforded a 23% RCC [23]. When the QMA preconditioning was carried out with 0.5 M K_2_CO_3_ (aq.), no radiolabeled compound could be detected. The combination of 0.5 M K_3_PO_4_ (aq.) and Bu_4_NOTf (20 mM in MeOH) elution conditions resulted in an RCC of 37 ± 4%. The higher valency of the phosphate anion combined with its high pKa (pKa = 12.7) is responsible for that. Multicharged anions with high pKa adhere more strongly to the resin, facilitating the fluorine-18 elution from the QMA with Bu_4_NOTf [21]. On the other hand, the base concentration is minimal within the eluted fraction reaction when the eluting anion chosen cannot displace the preconditioning anion. Consequently, only eluting anions are eluted within the process and preconditioning anions remained on the QMA. This dependency was recently studied in great detail by Bratteby et al. [21].

### 2.2. Influence of Methanol as a QMA Eluate

We decided to study the influence of methanol as an eluent in order to release trapped fluoride-18 from the QMA. Richarz et al. applied methanol for this purpose and showed that this approach could substantially reduce the required time needed for the elution process. The azeotropic distillation in particular could be accelerated, and 90–98% of the activity could be eluted from the QMA using methanol as an eluent [22]. Moreover, Zarrad et al. reported that Cu-mediated ^18^F-fluorination is able to tolerate relatively high amounts of alcohols. They speculate that this is related to the fact that solvation of fluorine with alcohols decreases its basicity, without compromising its nucleophilicity [19,29]. For this reason, the use of MeOH in combination with Bu_4_NOTf was tested. As expected, MeOH positively affected the EE of the trapped fluorine-18 (>90%) while eliminating the azeotropic drying step. The MeOH evaporation process, used for the drying of fluorine-18, takes 10 min at 100 °C. Therefore, the reaction time was reduced by 20 min, without compromising the efficiency of the reaction.

### 2.3. Precursor and Catalyst 

**Figure 3:**
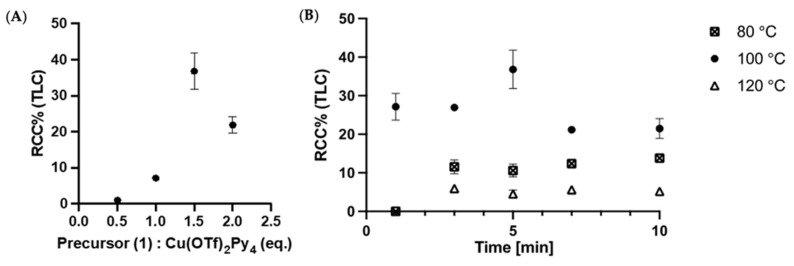
(**A**) Effect on RCC when adding different equivalents of Cu(OTf)_2_Py_4_ (0.5, 1, 1.5, 2) with respect to a fixated precursor amount (**2**). (**B**) Influence on RCC when changing time and temperature. Reaction conditions: PO_4_^3−^ QMA precondition, eluted with Bu_4_NOTf (20 µmol) in MeOH. Precursor **2** (3.2 mg, 10 µmol) and (**A**) different equivalent of Cu(OTf)_2_Py_4_ in DMA, for 5 min at 100 °C; (**B**) Cu(OTf)_2_Py_4_ (1.5 equiv., 10 mg, 15 µmol) for different time points (3, 5, 7, 10 min) and temperature (80 °C, 100 °C and 120 °C). The crude reaction was followed by radio-TLC and radio-HPLC (SI, Table S3). *n* = 3, error bars = standard deviation.

It is well known that precursor concentration and the precursor/catalyst ratio influence the RCC of Cu-mediated fluorinations. Therefore, we decided to investigate the dependency of these parameters on our model reaction. The solvent was set to DMA for all reactions, since the benefit of using an amide-based solvent in this reaction was already shown in previous works [18,19]. The best preconditioning and elution conditions identified in Section 2.1 were used. Furthermore, we decided to use Cu(OTf)_2_Py_4_ as a catalyst. This catalyst was reported to result in higher RCCs [19,30,31,32]. Different precursor/catalyst ratios were later investigated, increasing the catalyst equivalents from 0.5 to 2.0 (SI, Table S2). A precursor/catalyst ratio of 1:1.5 resulted in the highest RCC, at approximately 40% (Figure 3a). Finally, the precursor concentration was investigated. Totals of 5 µmol, 10 µmol, and 20 µmol (in 0.5 mL DMA) resulted in a 19%, 37%, and 9% RCC, respectively. These results showed that 10 µmol resulted in the best RCC.

### 2.4. Time and Temperature Optimisation

In order to identify the temperature and time point resulting in the highest RCC, three temperatures (80, 100 and 120 °C) were screened over a timeframe of 10 min. The resulting time–RCC curves are displayed in Figure 3B. The highest RCC was observed at 100 °C at 5 min. Higher and lower temperatures and different time points resulted in lower RCC. The lowered RCC after a reaction time of 5 min indicates that decomposition of the Tz is faster with the incorporation of fluoride-18 after 5 min. Moreover, decomposition appears to be faster at higher temperatures and ^18^F-incorporation lower at lower time and temperatures (SI, Table S3). 

### 2.5. Automated Synthesis of Cu-Mediated ^18^F-Fluorination Reaction

Optimized reaction conditions were translated to an automated synthesis module. Synthesis started from 10–12 GBq of [^18^F]fluoride received from the cyclotron, resulted in a 23 ± 3% RCY (d.c.), Am of 210 ± 15 GBq/µmol (dc), and RCP >98% of [^18^F]**1** (*n* = 2). A total amount of 1.2–1.6 GBq could be isolated at the end of the synthesis. The protocol was completed in a total synthesis time of 40 min, including [^18^F]fluoride drying, labeling, high performance liquid chromatography (HPLC) separation, and formulation (SI, Figures S3–S5)).

This protocol was then used to radiolabel [^18^F]**3** in order to show that identified radiolabeling conditions can be translated to other Tzs. [^18^F]**3** appears to be a good candidate as this tracer is our lead candidate to be translated to clinical applications. 

Initially, the labeling conditions were applied on the tert-butyl protected [^18^F]**3c**. Labeling of this structure resulted in an improved RCC of 40 ± 5% compared to previously applied conditions (Table 2). Therefore, we decided to automate the synthesis. The reaction was carried out in a two-step, one-pot sequence, resulting in an overall RCY of 14 ± 3%, Am of 201 ± 30 GBq/µmol (d.c.), and an RCP of ≥99% (*n* = 2) (including deprotection of [^18^F]**3c to** [^18^F]**3**). Total synthesis was completed in 70 min from received [^18^F]fluoride to formulated [^18^F]**3**. The activity yield was 1.3 to 2 GBq from ~12 GBq of fluoride-18 (Table 2, Figures S6–S8). This resulted in an improvement of 27%.

## 3. Experimental Section

### 3.1. Organic Synthesis

**Methods.** NMR spectra were acquired on a 600 MHz Bruker Avance III HD (600 MHz for ^1^H and 151 MHz for ^13^C), a 400 MHz Bruker Avance II (400 MHz for ^1^H, 376 MHz for ^19^F and 101 MHz for ^13^C) and a 400 MHz Bruker Avance UltraShield (400 MHz for ^1^H, 376 MHz for ^19^F and 101 MHz for ^13^C), using Chloroform-*d*, MeOD or DMSO-*d*_6_ as deuterated solvent and with the residual solvent as the internal reference. NMR spectra of all compounds were reprocessed in MestReNova software (version 12.0.22023) from original FID’s files. Yields refer to isolated compounds estimated to be >90% pure as determined by ^1^H NMR (25 °C) and analytical HPLC. Analytical HPLC method: Thermo Fisher UltiMate 3000 with a C-18 column (Luna 5 μm C18(2) 100 Å, 150 mm × 4.6 mm). Eluents: A, H_2_O with 0.1% TFA; B, MeCN with 0.1% TFA. Gradient from 100% A to 100% B over 12 min, back to 100% A over 3 min, flow rate 2 mL/min. Detection by UV absorption at λ = 254 nm on a UVD 170U detector. Thin-layer chromatography (TLC) was carried out on silica gel 60 F_254_ plates from Merck (Germany). Visualization was accomplished by UV lamp (254 nm). Preparative HPLC was carried out on an UltiMate HPLC system (Thermo Scientific, Waltham, MA, USA) consisting of an LPG-3200BX pump (10 mL/min), a Rheodyne 9725i injector, a 10 mL loop, a MWD-3000SD detector (254 nm), and an AFC-3000SD automated fraction collector, using a Gemini-NX C18 column (21.2 × 250 mm, 5 µm, 110Å) (Phenomenex) equipped with a guard. Purifications were performed using linear gradients of 0.1% TFA in MiliQ-H_2_O (A) and 0.1% TFA, 10% MiliQ-H_2_O in MeCN (B). Data were acquired and processed using Chromeleon Software v. 6.80. Semi-preparative HPLC was performed on the same system using a Luna 5µ C18 column (250 × 10 mm) with a flow rate of 3 mL/min. Automated Flash Column Chromatography was performed on a CombiFlash NextGen 300+ system supplied by TeleDyne ISCO, equipped with RediSep silica packed columns. Detection of the compounds was carried out by means of a UV-Vis variable wavelength detector operating from 200 to 800 nm and by Evaporative Light Scattering Detector (ELSD). Solvent systems for separation were particular for each compound but consisted of various mixtures of heptane, EtOAc, DCM and MeOH. Microwave-assisted synthesis was carried out in a Biotage Initiator apparatus operating in single mode, with the microwave cavity producing controlled irradiation at 2.45 GHz (Biotage AB, Uppsala, Sweden). The reactions were run in sealed vessels. These experiments were performed by employing magnetic stirring and a fixed hold time using variable power to reach (within 1–2 min) and then maintain the desired temperature in the vessel for the programmed time. The temperature was monitored by an IR sensor focused on a point on the reactor vial glass. The IR sensor was calibrated to internal solution reaction temperature by the manufacturer. Mass spectra analysis was completed using MS-Acquity-A: Waters Acquity UPLC with QDa-detector.

Materials. Unless otherwise stated, all reagents and solvents were purchased from commercial suppliers and used without further purification. All the water used was ultrapure (>18.2 MΩ cm^−^^1^). Other solvents were analytical or HPLC grade and used as received.

3-(3-Fluorophenyl)-1,2,4,5-tetrazine (**1**) was synthesized as previously described by Garcia-Vazquez et al. (Scheme S1) [23]. Rf = 0.34 (n-heptane:10%EtOAC); ^1^H NMR (400 MHz, Chloroform-d) δ 10.25 (s, 1H), 8.44 (dt, *J* = 7.8, 1.3 Hz, 1H), 8.33 (ddd, *J* = 9.7, 2.7, 1.6 Hz, 1H), 7.60 (td, *J* = 8.1, 5.7 Hz, 1H), 7.36 (tdd, *J* = 8.3, 2.7, 1.0 Hz, 1H); ^13^C NMR (101 MHz, Chloroform-d) δ 165.9 (d, *J* = 3.3 Hz), 163.5 (d, *J* = 247.6 Hz), 158.2, 133.9 (d, *J* = 8.2 Hz), 131.2 (d, *J* = 8.0 Hz), 124.2 (d, *J* = 3.2 Hz), 120.4 (d, *J* = 21.3 Hz), 115.3 (d, *J* = 24.1 Hz); ^19^F NMR (376 MHz, Chloroform-d) δ -107.77 (F); HPLC-MS [M^+^H]^+^ *m/z* calc. for [C_8_H_6_FN_4_]^+^: 177.05; Found: 177.54. 

3-(3-Trimethyltin)-1,2,4,5-tetrazine (**2**) was synthesized starting from 3-(3-iodophenyl)-1,2,4,5-tetrazine (**2a**), which was synthesized as previously described by Garcia-Vazquez et al. (Scheme S1) [23]. Rf = 0.30 (n-heptane:10%EtOAc); ^1^H NMR (400 MHz, Chloroform-d) δ 10.21 (s, 1H), 8.74 (d, *J* = 1.8 Hz, 1H), 8.55 (dt, *J* = 7.9, 1.6 Hz, 1H), 7.78 (d, *J* = 7.2 Hz, 1H), 7.57 (t, *J* = 7.5 Hz, 1H), 0.37 (s, 7H).; ^13^C NMR (101 MHz, Chloroform-d) δ 167.0, 157.9, 144.3, 140.8, 135.6, 131.1, 128.9, 128.3, -9.3.; HPLC-MS [M+H]+ *m/z* calc. for [C_11_H_15_SnN_4_]+: 323.04; Found: 323.38.

2,2′-((3-Fluoro-5-(1,2,4,5-tetrazin-3-yl)benzyl)azanediyl)diacetic acid (**3**) was synthesized as previously described by Garcia-Vazquez et al. as a TFA salt (Scheme S2) [23]. ^1^H NMR (400 MHz, Methanol-*d*_4_) δ 10.42 (s, 1H), 8.60 (d, *J* = 1.4 Hz, 1H), 8.41–8.32 (m, 1H), 7.73–7.64 (m, 1H), 4.59 (s, 2H), 4.11 (s, 4H); ^13^C NMR (101 MHz, Methanol-*d*_4_) δ 168.7, 165.1 (d, *J* = 3.2 Hz), 163.3 (d, *J* = 247.6 Hz), 158.2, 135.4 (d, *J* = 7.7 Hz), 135.1 (d, *J* = 8.6 Hz), 126.1 (d, *J* = 3.0 Hz), 121.7 (d, *J* = 22.7 Hz), 115.5 (d, *J* = 24.4 Hz), 57.9, 53.6; ^19^F NMR (376 MHz, Methanol-*d*_4_) δ -77.24 (TFA), -111.81 (F); HPLC-MS [M+H]^+^ *m/z* calc. for [C_13_H_13_FN_5_O_4_]^+^: 322.27; Found: 322.27.

Di-tert-butyl 2,2′-((3-(1,2,4,5-tetrazin-3-yl)-5-(trimethylstannyl)benzyl)azanediyl) diacetate (**4**) was synthesized as previously described by Garcia-Vazquez et al. (Scheme S2) [23]. *Rf* = 0.37 (n-heptane:20%EtOAc); ^1^H NMR (400 MHz, Chloroform-*d*) δ 10.20 (s, 1H), 8.63 (d, *J* = 1.8 Hz, 1H), 8.55 (s, 1H), 7.97–7.73 (m, 1H), 4.08 (s, 2H), 3.50 (s, 4H), 1.47 (s, 18H), 0.37 (s, 9H); ^13^C NMR (101 MHz, Chloroform-*d*) δ 170.1, 166.8, 157.7, 144.3, 141.4, 134.6, 131.1, 129.0, 81.3, 57.4, 55.1, 28.2, -9.3; HPLC-MS [M+H]^+^ *m/z* calc. for [C_24_H_38_N_5_SnO_4_]^+^: 580.19; Found: 580.22.

### 3.2. Radiochemistry

General information. At the Department of Clinical Physiology, Nuclear Medicine and PET, Rigshospitalet, Denmark, [^18^F]Fluoride was produced from (p,n)-reaction in a cyclotron (60 mikroA CTI Siemens or 40 mikroA Scanditronix) by irradiating [^18^O]H_2_O with a 11 MeV (CTI siemens) or 16 MeV (Scanditronix) proton beam. Automated syntheses were produced in a hot cell containing a Scansys Laboratorieteknik synthesis module. The Chromeleon software was used to execute the results. Analytical HPLC was achieved on a Dionex system connected to a P680A pump, a UVD 170U detector and a Scansys radiodetector. On the built-in HPLC system, Semi-preparative HPLC was executed in a synthesis module with a flow rate of 4 mL/min. Manual syntheses were produced in a hot cell. 

Radiochemical conversion (RCC) of all radiolabeled compounds was analyzed by radio-HPLC with an integration of the radioactive peaks from the reaction solution [33]. The radio-HPLC traces were compared to HPLC UV traces of the ^1^^8^F-reference sample for characterization. The radiochemical yield (RCY) was calculated from the activity given by the cyclotron and the formulated product at the end of the synthesis and has been decay corrected (d.c.). The molar activity (A*_m_*) was calculated from the integration area of the UV absorbance peak which corresponds to the radiolabeled product on the HPLC chromatogram. This area was converted to a molar mass by comparing it to an average of integrated areas (triplet) at known concentrations for the corresponding reference compounds. The radiochemical yield (RCY), radiochemical purity (RCP), and molar activity (Am) values are given as means. This is true for all the radiolabeled compounds listed below.

General procedure for the preparation of anhydrous [^18^F]fluoride for radiolabeling. An anion exchange resin (Sep-Pak Light Waters Accell Plus QMA cartridge, chloride form) was washed with EtOH (5 mL), precondition eluent (10 mL), H_2_O (10 mL) and dried with air. Aqueous [^18^F]fluoride solution was passed through the QMA (30–50 MBq), followed by 0.5 mL of MeOH. The QMA was eluted with 1 mL of the elution solution in MeOH. The wet [^18^F]fluoride was dried under a nitrogen stream at 100 °C for 5 min, to give no-carrier-added Bu_4_N[^18^F]F complex. 

Manual labeling of tetrazine [^18^F]1 starting from precursor **2**. A solution of organotin precursor (0.01 mmol) and Cu(OTf)_2_Py_4_ (1.5 equiv.) was dissolved in 0.5 mL DMA. This mixture was added to the dried Bu_4_N[^18^F]F and heated at 100 °C for 5 min. The mixture was cooled for 2 min before quenching with 1 mL of H_2_O/0.1%TFA. The syringe was flushed up and down once to elute the mixture and release any possible ^18^F from the vial walls. Samples were analyzed by radio-TLC eluting 0.05 mL of the crude in 0.1 mL H_2_O (samples activity conc.: 1–0.05 MBq/100 µL), using an eluent of heptane/EtOAc (50:50), rf = 0.6. Samples were analyzed by analytical-HPLC, by eluting 0.05 mL of the crude in 1 mL H_2_O/MeCN (50:50) (SI, Figures S1 and S2). Analytical HPLC method: Thermo Fisher UltiMate 3000 with a C-18 column (Luna 5 μm C18(2) 100 Å, 150 mm × 4.6 mm). Eluents: A, H_2_O with 0.1% TFA; B, MeCN with 0.1% TFA. Gradient from 100% A to 100% B over 12 min, back to 100% A over 3 min, flow rate 2 mL/min.

Automated labeling of tetrazine [^18^F]1 starting from precursor **2**. Automated synthesis was achieved on a Scansys Laboratorieteknik synthesis module. The optimized protocol was implemented as described above. The stannane precursor **2** (0.01 mmol) and Cu(OTf)_2_Py_4_ (1.5 equiv., 10.2 mg) in 0.5 mL DMA was added to a reaction vial containing the dried Bu_4_N[^18^F]F, and heated at 100 °C for 5 min. The crude mixture was then cooled down to 40 °C with compressed air, followed by quenching with 2 mL of H_2_O/0.1% TFA. The solution was purified via semi-preparative HPLC (Thermo Fisher UltiMate 3000) with a Luna 5 μm C-18(2) column (100 Å, 250 mm × 10 mm) using an isocratic method based on H_2_O/MeCN (40/60 *v*/*v*) and a flowrate of 4 mL/min. The collected fraction was dissolved in 40 mL water and passed through a Sep-Pak C18 Light (SPE) (previously precondition with 10 mL EtOH, 10 mL H_2_O and dried with air). The compound was recovered by eluting the SPE with 1 mL of EtOH into 0.1 M phosphate buffer (pH 7.4) for formulation in 10% EtOH/PBS. The automated protocol including drying of the wet [^18^F]fluoride, labelling, HPLC separation and formulation was completed within 40 min. Radio-HPLC and UV-HPLC of the formulated [^18^F]**1** product can be found in SI: Analytical Radio-HPLC and semi-preparative HPLC chromatograms of formulated [^18^F]Tzs.

Automated labeling of tetrazine [^18^F]3 starting from precursor **4**. 

A solution of precursor **4** (0.01 mmol) and Cu(OTf)_2_Py_4_ (1.5 equiv., 10.2 mg) in 0.5 mL DMA was added to a reaction vial containing the dried Bu_4_N[^18^F]F, and heated at 100 °C for 5 min. The crude mixture was then cooled down to 40 °C with compressed air, followed by quenching with 2 mL of H_2_O/0.1% TFA. The solution was then passed through a Sep-Pak C18 Plus cartridge preconditioned by flushing with 10 mL of EtOH, 10 mL of H_2_O and dried with air. The SPE was washed by flushing 10 mL H_2_O and dried with air, before eluting the [^18^F]tracer with 3 mL of MeCN into a reaction vial containing 1 mL of TFA. The solution was heated at 80 °C for 10 min, for deprotection of [^18^F]**3**. After full deprotection, the mixture was gently concentrated at 100 °C under an N_2_ stream for 20 min. The reactor vial was cooled to 40 °C, before the addition of 2.5 mL of H_2_O to resolubilize the mixture. The solution was then purified via semi-preparative HPLC (Thermo Fisher UltiMate 3000) with a Luna 5 μm C-18(2) column (100 Å, 250 mm × 10 mm) using an isocratic method based on 15% EtOH/H_2_O 0.1% TFA and a flowrate of 4 mL/min. The collected fraction was dissolved in 40 mL water and passed through a Sep-Pak C18 Light (SPE) (previously precondition with 10 mL EtOH, 10 mL H_2_O, and dried with air). The compound was recovered by eluting the SPE with 1 mL of EtOH into 0.1 M phosphate buffer (pH 7.4) for formulation in 10% EtOH/PBS. The automated protocol including drying of the wet [^18^F]fluoride, labelling, deprotection, HPLC separation, and formulation was completed within 70 min. Radio-HPLC and UV-HPLC of the formulated [^18^F]**3** product can be found in SI: Analytical radio-HPLC and semi-preparative HPLC chromatograms of formulated [^18^F]Tzs.

## 4. Conclusions

Herein, we described our successful efforts to optimize our recently developed strategy to ^18^F-label Tzs. The RCY of this Cu-mediated fluorination could be increased, optimizing the elution and identifying the best copper catalyst. Best reaction conditions resulted in an RCC of 37 ± 4% for [^18^F]**1** and an RCC of 40 ± 5% for [^18^F]**3c**. Hence, the RCC could be increased by a factor of 3 and 1.6, respectively (compared to the initially reported conditions). The implementation of these conditions was able to be translated to an automated synthesis module. This resulted in a 2.1-fold higher RCY for [^18^F]**1** with an Am of 210 ± 15 GBq/µmol and a RCP > 99%. Similarly, [^18^F]**3** could be radiolabeled in an RCY of 14 ± 3% (1.3-fold increase) with an Am of 201 ± 30 GBq/umol. In conclusion, we have identified a good starting point for optimization attempts to ^18^F-label highly reactive Tzs via Cu-mediated fluorinations and were able to increase the RCY of [^18^F]**3c**, our lead compound to be translated into the clinic.

## Figures and Tables

**Table 1:** Parameters explored for the optimization of the labeling of [^18^F]**1**.

Parameters Optimized	Conditions	Parameters Optimized	Conditions
**Preconditioning of** **the QMA** **with 10 mL of**	K_3_PO_4_ 0.5 M K_2_HPO_4_ 0.5 M KOTf 0.5 M K_2_CO_3_ 0.5 M	Catalyst equivalence Cu(OTf)_2_Py_4_ [eq.]	0.5 1 *1.5* 2
**Elution** (with 1 mL)	20 mM Bu_4_NOMs ^a^ 20 mM Et_4_NHCO_3_^a^ 50 mM KOTf/0.36 mM K_2_CO_3_^b^ 20 mM Bu_4_NOTf ^a^ 20 mM Bu_4_NH_2_PO_4_^a^ 150 mM TEAHF ^a^	Time [min]	3 *5* 7 10
**Precursor amount** [µmol] **in 0.5 mL** **DMA**	5 *10* 20	Temperature [°C]	80 *100* 120

^a^ Dissolved in 1 mL MeOH. ^b^ Dissolved in 0.55 mL H_2_O; bold and blue marked conditions resulted in the best radiochemical conversion; italic marked condition are the conditions previously identified to work best.

**Table 2:** Results of the Cu-mediated ^18^F-fluorination optimization for H-Tzs [^18^F]**1** and [^18^F]**3**.

Name	Structure	RCC (%) [23]	RCC (%) (This Work)	RCY (%) [23]	RCY (%) (This Work)	RCY Increased
[^18^F]**1**	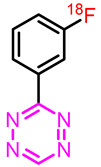	12 ± 1	37 ± 4	11 ± 3	23 ± 3	**10** **9%** **(factor 2.09)**
[^18^F]**3**	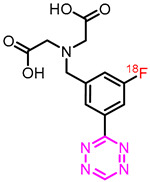	25 ± 4 ^a^	40 ± 5 ^a^	11 ± 3	14 ± 3	**23%** **(factor 1.3)**

^a^ This value correspond to the Cu-mediated 18-fluorinated reaction of [^18^F]**3c** tert-butyl protected. For all the reactions *n* = 3 and error bars = standard deviations.

## Data Availability

The data presented in this study are available in Supplementary Material.

## Supplementary Materials

The following are available online at https://www.mdpi.com/article/10.3390/molecules27134022/s1, Scheme S1: Synthesis of Tzs [^18^F]1; Scheme S2: Synthesis of Tzs [^18^F]3; Table S1: Optimization of different preconditioning and elution solvents; Table S2: Optimization of precursor and catalyst amount. The crude reaction was followed by radio-TLC and radio-HPLC; Table S3: Optimization of time and temperature. The crude reaction was followed by radio-TLC and radio-HPLC; Figure S1: Radio-HPLC chromatogram of crude mixture of [^18^F]1 (Rt = 7.280 min). Reaction conditions: [^18^F]KF, Cu(OTf)2, pyridine, DMA, 5 min, 100 °C, 5.92 % RCC ([^18^F]3); Figure S2: Analytical-HPLC chromatogram of reference compound 3 (UV/Vis, 254 nm) and radio-HPLC chromatogram of crude mixture of [^18^F]1 (Rt = 7.227 min). Reaction conditions: [^18^F]Bu4NF, Cu(OTf)2Py4, DMA, 5 min, 100 °C, 34.2 % RCC ([^18^F]3); Figure S3: Semi-preparative HPLC chromatogram for [^18^F]1 (Rt = 790 s); Figure S4: Analytical-HPLC chromatogram of reference compound 1 (UV/Vis, 254 nm) and radio-HPLC chromatogram of formulated [^18^F]1 (Rt = 6.87 min). The solid red line indicates the radio-HPLC [^18^F]tracer and the solid black line indicates the UV trace for the cold reference compound; Figure S5: Radio-HPLC chromatogram of formulated [^18^F]1 (Rt = 6.87 min) black line, followed by UV_VIS spectra 220 nm (blue), 254 (magenta), 290 nm (green) and 320 nm (orange).Figure S6: Semi-preparative HPLC chromatogram for [^18^F]3 (Rt = 580 s); Figure S7: Analytical-HPLC chromatogram of reference compound 3 (UV/Vis, 254 nm) and radio-HPLC chromatogram of formulated [^18^F]3 (Rt = 4.86 min); Figure S8: Radio-HPLC chromatogram of formulated [^18^F]3 (Rt = 4.86 min) black line, followed by UV_VIS spectra 220 nm (blue), 254 nm (magenta), 290 nm (green) and 320 nm (orange) [23].

## Sample Availability

Not available.
