# Supplementary material for: Optimization of Direct Aromatic 18F-Labeling of Tetrazines
Source: Molecules. 2022 Jun 22;27(13):4022. doi: 10.3390/molecules27134022 (PMC9268649; doi:10.3390/molecules27134022)
Supplement: Supplementary file 1 [file molecules-27-04022-s001.zip › molecules-1738980-supplementary.pdf]

Supporting Information (SI)

Optimization of Direct Aromatic ^{18}F-Labeling of Tetrazines

Ida Vang Andersen [1,2,†], Rocío García-Vázquez [1,2,†], Umberto M. Battisti [1] and Matthias M. Herth [1,2,*]

[1] Department of Drug Design and Pharmacology, Faculty of Health and Medical Sciences, University of Copenhagen, Jagtvej 160, 2100 Copenhagen, Denmark; ida.andersen@sund.ku.dk (I.V.A.); rociogv@sund.ku.dk (R.G.-V.); umberto.battisti@sund.ku.dk (U.M.B.)
[2] Department of Clinical Physiology, Nuclear Medicine & PET, Rigshospitalet, Blegdamsvej 9, 2100 Copenhagen, Denmark
* Correspondence: matthias.herth@sund.ku.dk
† These authors contributed equally to this work

Table of Contents

General reaction scheme 2

Radiochemistry 3

Analytical Radio-HPLC and semi-preparative HPLC chromatograms of formulated [^{18}F]Tzs 4

Chromatograms of manual synthesis 4

Chromatograms of automated synthesis 5

General reaction scheme

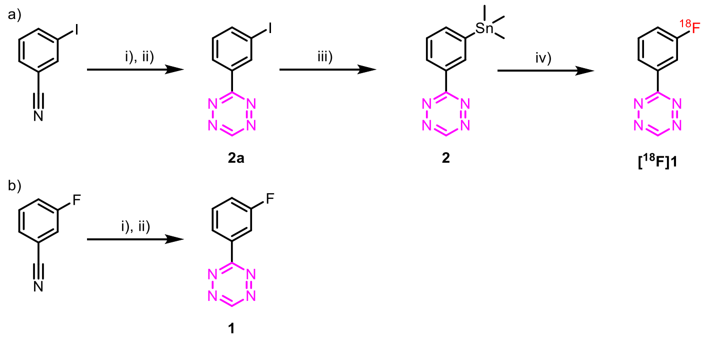

Scheme S1. Synthesis of Tzs [^{18}F]**1** (a) and **1** (b). i) DCM, S_8, $NH_2NH_2 \cdot H_2O$, EtOH, 50 ºC, 24 h; ii) $NaNO_2$, AcOH, 0 ºC, 20 min, 33% (**2**), 34% (**1**); iii) $(Me_3Sn)_2$, $Pd(OAc)_2$, ^{me}CgPPh, THF, 70 ºC, MW, 30 min., 58% (**3**); iv) [^{18}F]Bu_4NF, $Cu(OTf)_2Py_4$, DMA, 5 min, 100 °C, 37% RCC ([^{18}F]**1**).

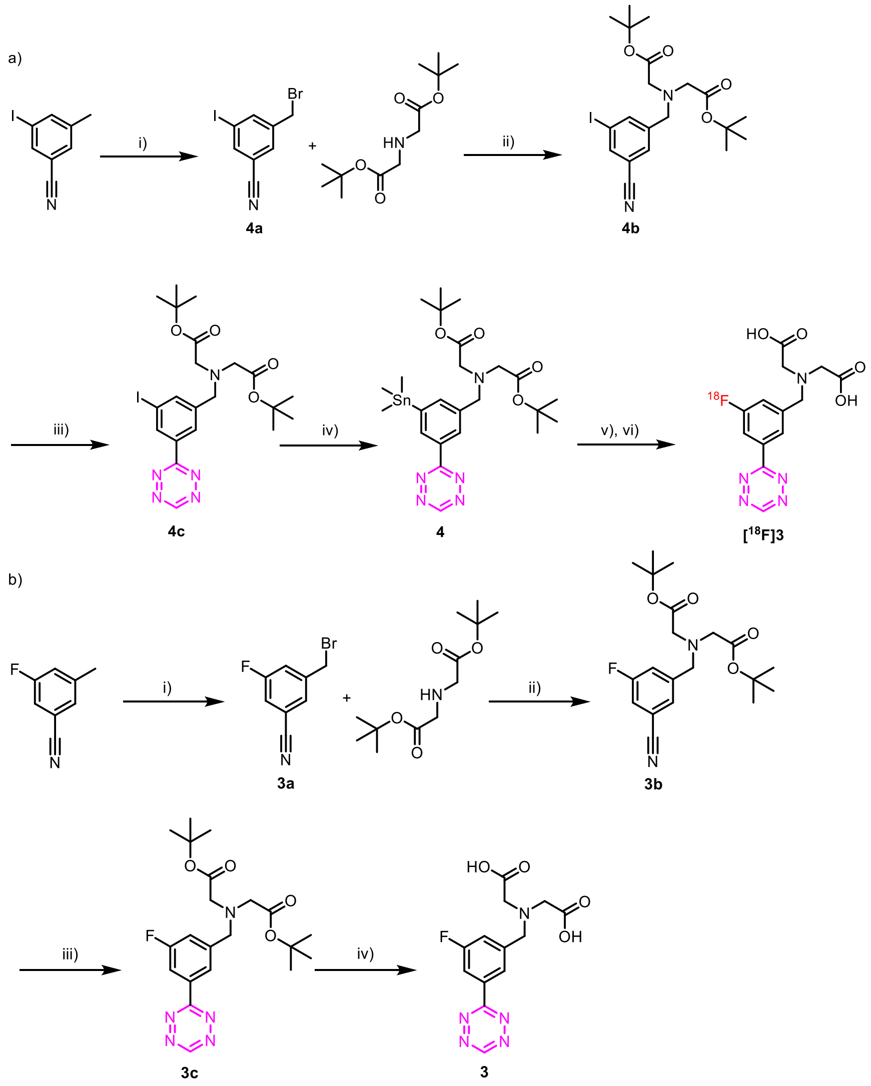

Scheme S2. Synthesis of Tzs [^{18}F]**3** (a) and **3** (b). **(a)** i) NBS, AIBN, $CHCl_3$, 65 ºC, 24 h; 49%; ii) K_2CO_3, MeCN, 25 ºC, 24 h, 99 %; iii) DCM, S_8, $NH_2NH_2 \cdot H_2O$, EtOH, 50 ºC, 24 h, 15–24%; iv) $(Me_3Sn)_2$, $Pd(PPh_3)_4$, THF, 65 ºC, MW, 3 h, 62%; v) $Cu(OTf)_2Py_4$, [^{18}F]Bu_4NF, DMA, 5 min, 100 °C; vi) TFA, MeCN, 100 ºC, 15 min, 14% RCY; **(b)** i) NBS, AIBN, $CHCl_3$, 65 ºC,

12 h, 52%; ii) K_2CO_3, MeCN, r.t., 24 h, 89%; iii) DCM, S_8, $NH_2NH_2 \cdot H_2O$, EtOH, 50 ºC, 24 h, 17%; iv) TFA, DCM, 25 ºC, 2 h, 51%.

Radiochemistry

Table S1. Optimization of different preconditioning and elution solvents. The crude reaction was followed by radio-TLC. Reaction conditions: QMA preconditioned, [^{18}F]fluoride (~50 MBq) loaded, flushed with 0.5 mL MeOH and eluted with one of the eluents shown in the table. Dried under nitrogen gas for 5 min at 100 °C before adding precursor **2** (3.2 mg, 10 µmol) and $Cu(OTf_2)Py_4$ (10 mg, 15 µmol) in 0.5 mL DMA, 5 min at 100 °C.

**Precondition**	Bu_4NOMs [2]	Et_4NHCO_3 [2]	$KOTf/K_2CO_3$ [1]	Bu_4NOTf [2]	$Bu_4NH_2PO_4$ [2]	TEAHF [2]
PO_4^{3-}	15.9 ± 5	17.8 ± 4	0	36.8 ± 5	5.9 ± 3	0
HPO_4^{2-}	1.5	-	-	1.8	-	-
OTf^-	-	-	23.3 ± 6	0	-	-
CO_3^{2-}	0	-	-	0	-	-

[1] Dissolved in 1 mL MeOH. [2] Dissolved in 1 mL H_2O. RCC% is measured with radio-TLC

Table S2. Optimization of precursor and catalyst amount. The crude reaction was followed by radio-TLC and radio-HPLC. Reaction conditions: PO_4^{3-} QMA precondition, eluted with Bu_4NOTf (20 µmol) in MeOH. Precursor **2** (3.2 mg, 10 µmol) and different equivalent of Cu(II) source in DMA, for 5 min at 100 °C.

**Precondition**	**Elution**	**Precursor : Cu(II) source (eq)**	**RCC% (TLC)**	**RCC% (HPLC)**
**OTf^-**	$KOTf/K_2CO_3$	1:2:15 [a]	11.9 ± 2	8.9 ± 4
**OTf^-**	$KOTf/K_2CO_3$	1:1.5 [b]	23.2 ± 7	21.4 ± 1
**PO_4^{3-}**	Bu_4NOTf	1:0.5 [b]	1.1 ± 0.1	-
**PO_4^{3-}**	Bu_4NOTf	1:1 [b]	7.2 ± 0.4	5 ± 0.2
**PO_4^{3-}**	Bu_4NOTf	1:1.5 [b]	36.8 ± 5	38.6 ± 2
**PO_4^{3-}**	Bu_4NOTf	1:2 [b]	21.9 ± 2	26.1 ± 4

[a] $Cu(OTf)_2$ and Pyridine equiv. , method from Garcia et al. 2021 [1]. [b] $Cu(OTf)_2Py_4$ equiv.

Table S3. Optimization of time and temperature. The crude reaction was followed by radio-TLC and radio-HPLC. Reaction conditions: PO_4^{3-} QMA precondition, eluted with Bu_4NOTf (20 µmol) in MeOH. Precursor **2** (3.2 mg, 10 µmol), $Cu(OTf)_2Py_4$ (1.5 equiv., 10 mg, 15 µmol) for different time points (3, 5, 7, 10 min) and temperature (80 °C, 100 °C and 120 °C).

**RCC%**	**80 °C**		**100 °C**		**120 °C**	
**Time [min]**	TLC	HPLC	TLC	HPLC	TLC	HPLC
**1**	0	0	27.1 ± 3	24.3 ± 0.4	-	-
**3**	11.5 ± 2	9.7 ± 1	27 ± 0.7	23.9 ± 1	6 ± 0.4	3
**5**	10.6 ± 2	12.1 ± 4	36.8 ± 5	38.6 ± 2	4.5 ± 0.5	4.7
**7**	12.4 ± 0.1	10 ± 0.8	26.2 ± 0.1	23.6 ± 2	5.6	4.8
**10**	13.8	4.7 ± 0.2	21.5 ± 3	13.8 ± 4	5.2	2.2

Analytical Radio-HPLC and semi-preparative HPLC chromatograms of formulated $[^{18}F]$Tzs

Chromatograms of manual synthesis

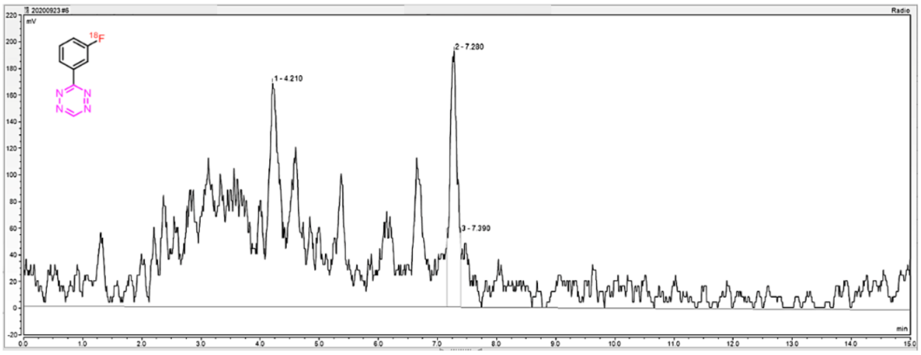

Integration Result				
No.	Retention Time [min]	Area mV·min	Height [mV]	Relative Area [%]
1	4.210	325.8	167.88	72.55
2	7.280	26.582	192.13	5.92
3	7.390	96.707	56.02	21.53
Total		449.08	416.04	100.00

Figure S1. Radio-HPLC chromatogram of crude mixture of $[^{18}F]$**1** (Rt = 7.280 min). Reaction conditions: $[^{18}F]$KF, $Cu(OTf)_2$, pyridine, , DMA, 5 min, 100 °C, 5.92 % RCC ($[^{18}F]$**3**).

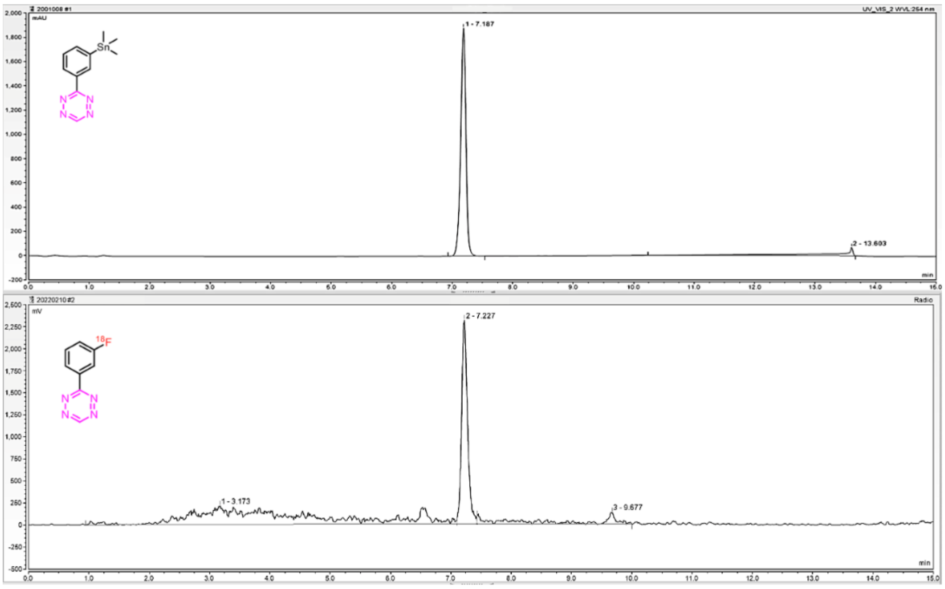

Integration Result				
No.	Retention Time [min]	Area mV·min	Height [mV]	Relative Area [%]
1	3.173	431.578	210.7	56.4
2	7.227	261.329	2309.49	34.2
3	9.677	72.258	134.57	9.4
Total		765.165	2654.76	100.00

Figure S2. Analytical-HPLC chromatogram of reference compound **3** (UV/Vis, 254 nm) and radio-HPLC chromatogram of crude mixture of $[^{18}F]$**1** (Rt = 7.227 min). Reaction conditions: $[^{18}F]Bu_4NF$, $Cu(OTf)_2Py_4$, DMA, 5 min, 100 °C, 34.2 % RCC ($[^{18}F]$**3**).

Chromatograms of automated synthesis

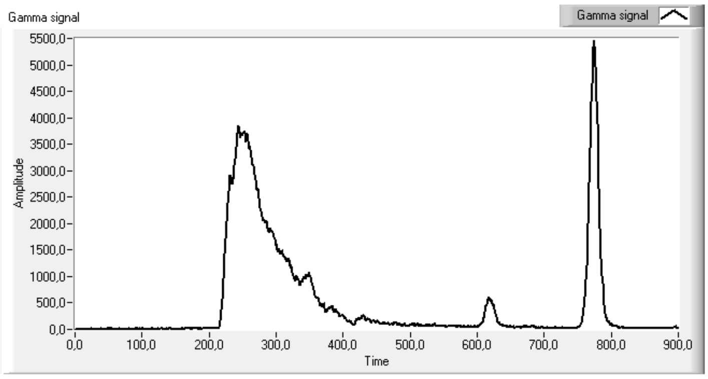

Figure S3. Semi-preparative HPLC chromatogram for **[^{18}F]1** (R_t = 790 s).

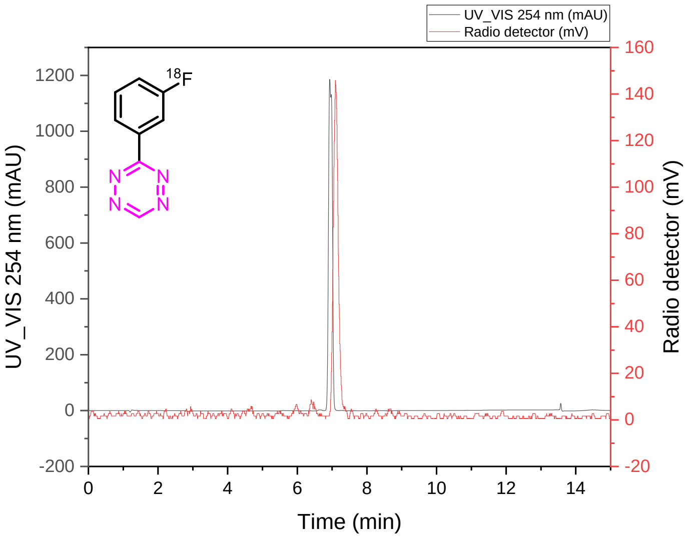

Figure S4. Analytical-HPLC chromatogram of reference compound **1** (UV/Vis, 254 nm) and radio-HPLC chromatogram of formulated **[**[18]F**]1** (Rt = 6.87 min). The solid red line indicates the radio-HPLC [^{18}F]tracer and the solid black line indicates the UV trace for the cold reference compound.

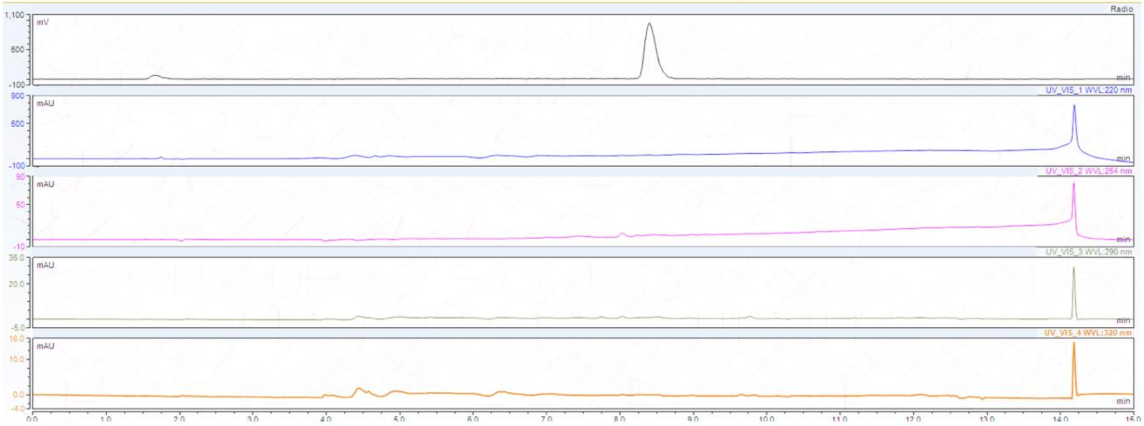

Figure S5. Radio-HPLC chromatogram of formulated [^{18}F]**1** (Rt = 6.87 min) black line, followed by UV_VIS spectra 220 nm (blue), 254 (magenta), 290 nm (green) and 320 nm (orange).

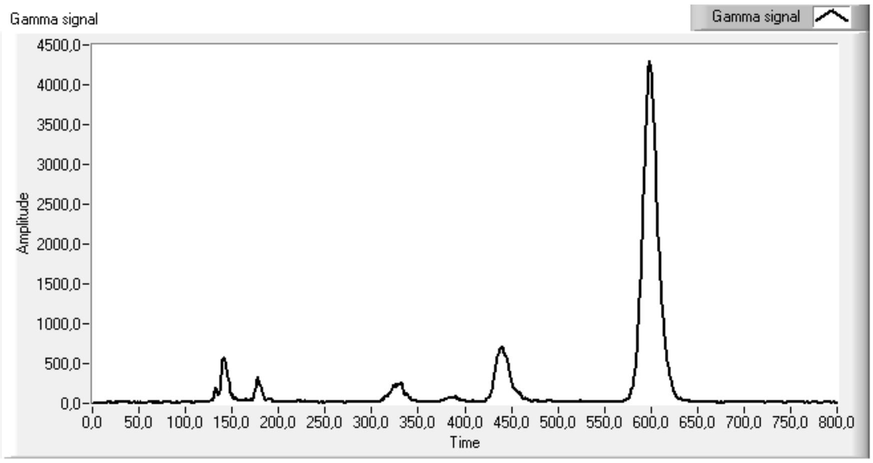

Figure S6. Semi-preparative HPLC chromatogram for **[^{18}F]3** (R_t = 580 s)

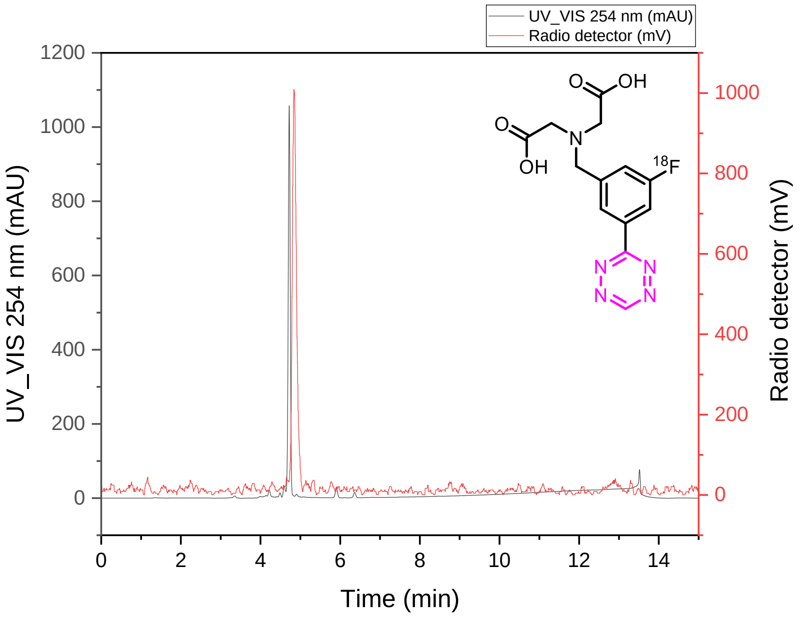

Figure S7. Analytical-HPLC chromatogram of reference compound **3** (UV/Vis, 254 nm) and radio-HPLC chromatogram of formulated **[^{18}F]3** (Rt = 4.86 min).

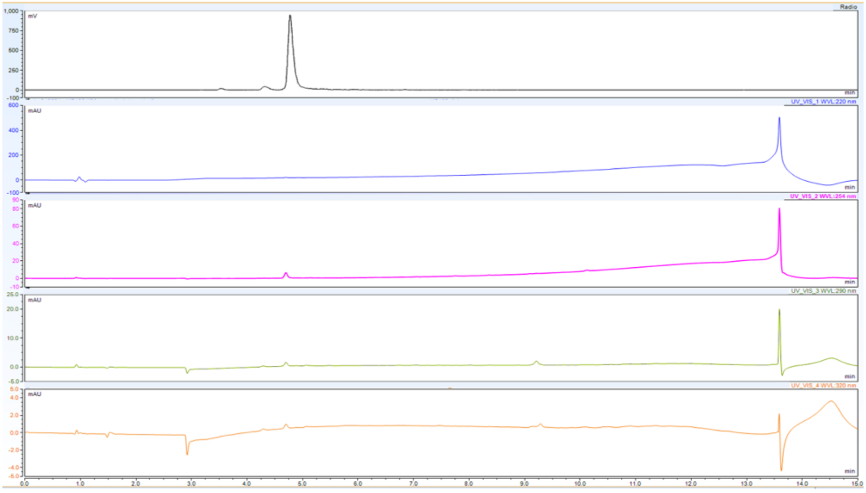

Figure S8. Radio-HPLC chromatogram of formulated **[**^{18}F**]3** (Rt = 4.86 min). black line, followed by UV_VIS spectra 220 nm (blue), 254 nm (magenta), 290 nm (green) and 320 nm (orange).
